# A Wearable Sensor-Based Platform for Surgeon Posture Monitoring: A Tool to Prevent Musculoskeletal Disorders

**DOI:** 10.3390/ijerph18073734

**Published:** 2021-04-02

**Authors:** Nicola Carbonaro, Gabriele Mascherini, Ilenia Bartolini, Maria Novella Ringressi, Antonio Taddei, Alessandro Tognetti, Nicola Vanello

**Affiliations:** 1 Department of Information Engineering, University of Pisa, 56122 Pisa, Italy; alessandro.tognetti@unipi.it (A.T.); nicola.vanello@unipi.it (N.V.); 2 Research Center ‘‘E. Piaggio,’’ University of Pisa, 56122 Pisa, Italy; 3 Department of Experimental and Clinical Medicine, University of the Study of Florence, 50121 Florence, Italy; gabriele.mascherini@unifi.it (G.M.); ilenia.bartolini@unifi.it (I.B.); marianovella.ringressi@unifi.it (M.N.R.); antonio.taddei@unifi.it (A.T.)

**Keywords:** ergonomic risk, IMU, musculoskeletal disease, RULA approach, spine posture, surgeon posture, wearable sensor

## Abstract

Surgeons are workers that are particularly prone to the development of musculoskeletal disorders. Recent advances in surgical interventions, such as laparoscopic procedures, have caused a worsening of the scenario, given the harmful static postures that have to be kept for long periods. In this paper, we present a sensor-based platform specifically aimed at monitoring the posture during actual surgical operations. The proposed system adopts a limited number of Inertial Measurement Units (IMUs) to obtain information about spine and neck angles across time. Such a system merges the reliability of sensor-based approaches and the validity of state-of-the-art scoring procedure, such as RULA. Specifically, three IMUs are used to estimate the flexion, lateral bending, and twisting angles of spine and neck. An ergonomic risk index is thus estimated in a time varying fashion borrowing relevant features from the RULA scoring system. The detailed functioning of the proposed systems is introduced, and the assessment results related to a real surgical procedure, consisting of a laparoscopy and mini-laparotomy sections, are shown and discussed. In the exemplary case study introduced, the surgeon kept a high score, indicating the need for an intervention on the working procedures, for a large time fraction. The system allows separately analyzing the contribution of spine and neck, also specifying the angle configuration. It is shown how the proposed approach can provide further information, as related to dynamical analysis, which could be used to enlarge the features taken into account by currently available approaches for ergonomic risk assessment. The proposed system could be adopted both for training purposes, as well as for alerting surgeons during actual surgical operations.

## 1. Introduction

Musculoskeletal Disorders (MSDs) in workers are a group of diseases affecting muscles, tendons, and nerves and covering a heterogeneous range of health conditions such as lower back pain and upper or lower limb injuries, which have a big impact on the quality of life and productivity [1,2,3]. In the case of workers employed in the health sector, there are relevant implications in terms of the quality of healthcare and patient outcomes [4]. Specifically, surgeons represent a category of workers that are particularly exposed to the risk of developing MSDs during their professional career in comparison with the general population [5]. It has been reported by many cross-sectional studies that up to 80% of physicians, including surgeons and interventionists, manifest evident pain during the surgical procedures, while the prevalence of injuries to the lumbar or cervical spine ranges between 17% and 19% along with the career, with an increase of the observed prevalence over the years [6]. Moreover, the occurrence of these injuries seems to be underestimated given the high percentage of workers (up to 80%) that have been screened who did not officially report such problems to their institution [4,5]. Surgeons are exposed to the risk of developing MSDs because of the physical stress consisting of repetitive movements and the assumption of uncomfortable and risky static postures during lengthy operations [7]. Specifically, risky static postures of spine, defined as that condition in which the head and upper back are flexed forward [8], characterize currently adopted surgical techniques such as laparoscopic procedures.

Laparoscopic surgery brought considerable advantages to the patients, including the reduction of postoperative pain and recovery time [9]. From the operators’ point of view, several studies have shown the high ergonomic risks due to the regular practice of laparoscopic surgery, regardless of the characteristics associated with the surgeon such as experience, sex, or age [10]. In fact, during laparoscopic surgery, the surgeon assumes static positions that are more harmful than those adopted during open surgery [11,12]. The limitation to the surgeon’s posture imposed by the trocars’ position on the patients and the handling of the laparoscopic instruments are some of the causes of these differences and may induce more muscular strain and fatigue [11,12]. Moreover, the visualization of the area of interest projected on the monitor, with a two-dimensional view, requires very fine eye-hand coordination and further constrains postures and movements. The spinal column is among the most affected body regions across its different sections such as cervical, thoracic, and lumbar. These musculoskeletal disorders, in the long run, may cause a decrease in surgical performance, with a consequent potential impact on patient safety and on the duration of the professional career of the surgeons [13,14].

To analyze such a scenario in order to identify and reduce such problems, it would be useful to employ ergonomic risk assessment procedures [15,16]. Specifically, such procedures can be adopted to inform the workers about the quality of the performed movements and postures assumed during the accomplished procedures and to help them to identify dangerous combinations of them. This information can be used to develop targeted ergonomics educational programs [17], to identify the subject-specific habits during the training phase, as well as during the actual procedure to promptly alert the surgeon. In general, ergonomic risk assessment methods can be classified into three different approaches: (1) the self-assessment method, in which data are collected by asking workers to answer interviews or questionnaires by filling in specific forms; (2) the semi-direct observational approach, in which professionals observe the execution of the worker’s activity and carry out a postural analysis using pre-filled forms; (3) direct methods, where the postures assumed and the various activities performed are recorded through the use of instruments and sensors positioned directly on the worker’s body [18]. The semi-direct methods have a long application history and are well described in the literature. Specifically, several tools were developed and optimized for different scenarios; some methods can be applied to general workload conditions; others are more focused on upper or lower limb assessment [15]. Moreover, the different methods can also be classified according to the cause of MSDs, which is the main focus of the evaluation step, as repetitive movements, strained posture, or handling of loads [16].

Considering the direct methods group, camera systems and wearable sensors represent the principal measuring devices used in the recent scientific literature for ergonomic operator assessment. Taking into account our goal to monitor surgeon spinal posture during a real operation, camera-based methods were not considered due to the limitations that the scenario offered such as scarce light conditions and the possibility of occlusion. On the other hand, wearable devices and in particular Inertial Measurement Units (IMUs), almost considered as the gold standard for body segment orientation estimation and joint angle extraction [19], have a small size and the capability to not obstruct or limit surgeon activity. In the study of Abyarjoo et al. [20], a wearable system using an IMU attached to the subject’s upper back was developed and tested to evaluate office workers’ posture in order to provide the indication of good postural habits. The study conducted by Yan et al. [21] concerned instead the use of IMUs for the analysis of spinal and neck movements in construction company workers, who are among the most at risk for the development of musculoskeletal pathologies. The developed system, tested in a laboratory setting simulating the real scenario, included an IMU positioned in the operator’s helmet and another positioned in the upper part of the back that send data via Bluetooth to a smartphone that in real time can alert the operator of possible risky movements. Vignais et al. in 2013 [22] developed a system capable of evaluating in real time the ergonomic risk of the worker performing manual tasks in an industrial environment through the Rapid Upper Limb Assessment (RULA) semi-direct method [23]. The measuring platform was composed by two goniometers synchronized with seven IMUs in order to reconstruct trunk, head, and arm movements. Moreover a see-through head-mounted display was used to augment the information provided to the user about his/her posture. The Sitting Posture Monitor (SPoMo) [24] is a system based on two IMU sensors positioned on the individual’s back (one on the lower part, the other on the upper one) useful for monitoring the worker’s posture and providing suggestions to prevent MSDs caused by improper postures of the spinal column during sedentary work. This system is able to extract the angular values assumed by spine, and when they exceed a predefined threshold value, the user is notified by a vibration mechanism that warns him/her of an incorrect and risky posture. Cerqueira et al. proposed in their study [25] a smart vest, which, in addition to monitoring the posture of the subjects, allowed generating in real time biofeedback to the worker through a vibro-tactile stimulus in order to immediately correct the posture. The system, consisting of three IMUs for the reconstruction of back and arm movements, allowed evaluating the ergonomic risk condition through the use of the RULA and Loading on Upper Body Assessment (LUBA) [26] semi-direct methods.

All these studies have in common the use of different IMUs distributed on the subject’s body to assess the posture of the worker, but most of these systems have been tested in laboratory environments simulating the working condition of the operator or applied in work contexts such as offices or building construction. In this work, we present and preliminarily evaluate a wearable platform that is well adapted to the surgeon’s working conditions, including a minimum set of IMUs and ease of integration in order to satisfy surgical field sterility requirements. One goal was to generate objective measures of the surgeon’s posture to identify potential ergonomic problems during the actual intervention without affecting the surgeons’ movement capabilities.

The proposed approach merges the movement and posture estimation approach of the direct methods and the validity of semi-direct methods, which have been evaluated in different scenarios [15]. The platform monitors spine and neck positions over time, given the relevance of this information for the assessment of ergonomic risk in surgeons. In this paper, we describe the setup procedures and the algorithm developed to estimate an ergonomic risk index, over time. The algorithm borrows relevant features and parameters from the well-established RULA method [23]. RULA evaluates the biomechanical and postural risk of the human body by analyzing mainly the behavior of neck, trunk, and upper limbs. The system capabilities are described using data acquired during a real surgical procedure.

## 2. Materials and Methods

### 2.1. RULA Assessment Tool

**Figure 1:**
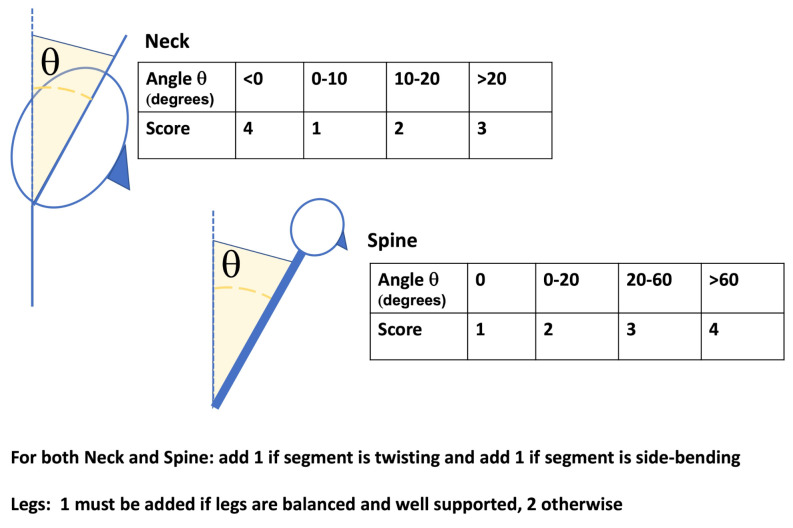
Representation of the posture evaluation of neck and trunk reported in section “B” of the RULA method. Figure modified from the work of McAtamney and Corlett [23].

RULA belongs to the semi-direct methods used for the identification of risks related to the onset of MSDs [23]. RULA is one of the first tools developed for the assessment of the ergonomic risk of the worker and the most commonly used and cited method to date. RULA is suitable to evaluate posture and static action [15]. It was shown to have a good inter-observer reproducibility and a good correspondence to other approaches such as Rapid Entire Body Assessment (REBA) [15]. RULA’s scoring procedure takes into account specific angles of body segments, and it can be easily adapted to a direct approach, when the corresponding angles are available [22], or used as a comparison for the validation of wearable sensor-based systems [27]. In its original application, RULA does not require special equipment and/or tools for its estimation and is based on the visual analysis of postures, muscular activities, and external loads to which some regions of the body of the worker are subjected. Specifically, the method analyzes neck, trunk, and upper limbs. Through a pre-printed questionnaire, the observer evaluates the posture of the worker, the forces exerted during the activity, and possible repetitions of harmful positions or movements. For each region of the body (upper arm, lower arm, wrist, neck, trunk, and legs) the evaluator assigns a score. Two partial scores are obtained, namely A and B. The former is related to arms and wrists, while the latter comprises neck, trunk, and legs (see Figure 1). Moreover, scores taking into account the muscle activity, whether static or repeated, and the load or the applied force are added. Once the data are collected and classified, the tables, available in the proforma sheets, allow generating a final score that represents the MSD risk level. The final index can assume values between 1 and 7, where 1 indicates a low level of risk, while 7 indicates a very high level of risk to develop musculoskeletal pathologies and, therefore, the need for intervention to prevent workers from having problems. Specifically, a score equal to 3 or 4 indicates that an intervention or change to the working procedure might be needed; a score equal to 5 or 6 indicates that changes are needed soon; while a score of 7 indicates an urgent need for a change in the working procedures.

**Figure 2:**
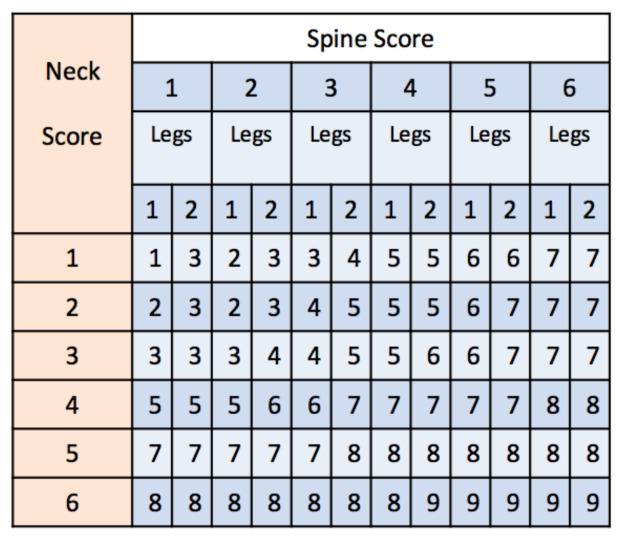
Look-up table to obtain the overall score for head and trunk. Leg information was entered as a constant (value equal to 1), given the specific scenario of this work.

In this study, we performed a joint angle measurement to identify the surgeon’s trunk and neck posture, in order to calculate the score of each body area, in particular to extract the “local Score B”, as represented in the RULA sheet. This local score merges the contributions of neck and spine using also the information about leg involvement: with both legs and feet in a supported and evenly balanced position, 1 must be taken into account, 2 otherwise (see Figure 2). According to the RULA scoring system [23], a local score related to neck, trunk, and leg, here indicated as “B”, equal to or greater than 6, constrains the final score of the complete RULA approach to be equal to or greater than 5, thus strongly indicating a needed change of the working procedure. A local score equal to 5 constrains the total score to be greater than 4. A local score equal to 3 or 4 constrains the total score of the complete RULA approach to be greater than 3, suggesting considering an intervention to reduce the risk of injury.

The method we present borrows some features from the RULA approach for scoring neck and trunk positions. Different from the RULA index, where a score is obtained for the entire working procedure, the algorithm we developed can be used to estimate a score, which we indicate as the ergonomic risk index, in a time-varying fashion.

### 2.2. Wearable Platform Developed

**Figure 3:**
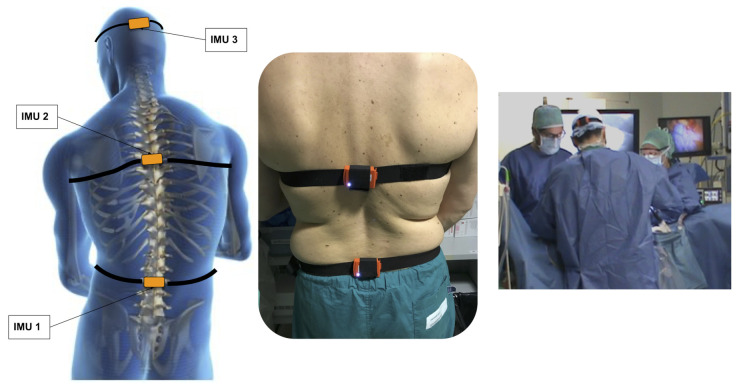
Representation of sensor placement, final application on the surgeon’s body, and an image of the Operating Room during the platform test.

To evaluate the variation of surgeon posture related to spine and neck movements, three IMUs were positioned in direct contact with the surgeon’s body. The IMUs selected were the MTw produced by Xsens [28]. These sensors are commonly considered a reference system for their robustness and accuracy in the 3D reconstruction of the motion and orientation of human body segments. The MTw sensors integrate 3D accelerometers, 3D gyroscopes, and 3D magnetometers and can supply both the raw values of the single elements and the orientation in the form of quaternions, Euler angles, or rotation matrices using proprietary fusion algorithms [29]. The IMU sensors have a dynamic accuracy of about 2 deg RMS and an angular resolution of 0.05 deg, as reported by the manufacturer. Each MTw transmits data to the Awinda Station, a dedicated hub for data synchronization and sensor charging, which is directly connected to the host PC using a USB port. The Awinda Station guarantees the time synchronization of the devices connected through the wireless network, and the MTw buffered data are made available to the host (PC). Finally, these sensors have a battery with a declared autonomy of about 3 h, which allows monitoring most laparoscopic surgery sessions [30]. Moreover, the processing of the output data from the MTw sensors allows us to reconstruct the rotation angle with an error of about 3 degrees [31,32]. In our study, sensor placement was conceived by taking into account the compromise between minimum number of sensors and platform performances on the extraction of trunk and neck angle variation. Moreover, the sensor platform design derives also from the consideration that in the observational methods for the estimation of ergonomics, the movement of the back, such as for neck, is associated with a single-segment biomechanical model. Consequently, considering for example spine as a single joint segment, trunk angle can be calculated from a single IMU commonly placed either on the chest [22] or on the upper back [21,25] or through 2 IMUs positioned respectively at the sacral level (as a body reference) and the other at the mid-thoracic level [33]. Our selection was based on this last configuration; in fact, the first sensor was placed in correspondence with the sacral level (IMU1), the second at the thoracic level (IMU2), and the last in the head region (IMU3) (see Figure 3). In this application, the IMUs platform was set to acquire a rotation matrix from each MTw sensor with a sampling frequency of 75 Hz. Before starting the surgical procedure, a baseline acquisition was performed, while the surgeon was standing upright in the anatomical position for 5 s. This position is considered to be the neutral posture in which head and upper trunk are aligned to the rest of spine. With this easy-to-implement calibration stage, the transformation matrix used to estimate the body segment orientation was then calculated by matching the sensor orientations in the global frame with the known orientations of each IMU [34]. Once the surgeon has started the surgical procedure, the angles produced by spine and neck are evaluated considering the current posture with respect to the one stored in the calibration phase.

(1) $$R_{GX_{new}}=R_{GX_{0}}^{-1}*R_{GX}$$

In particular, the information related to the spine angle variation is calculated using the IMU2 rotation matrix ($R_{IMU2}$) with respect to the ones of IMU1 ($R_{IMU1}$). As regards neck monitoring, the angles are estimated using the rotation matrices of IMU3 ($R_{IMU3}$) compared to those collected by IMU2.

(2) $$R_{Spine}=R_{IMU1}^{-1}*R_{IMU2}$$

(3) $$R_{Neck}=R_{IMU2}^{-1}*R_{IMU3}$$

Using this configuration, it is possible to detect the angles assumed by the desired body segments over time [31,35]. These values are used to calculate the ergonomic risk index, such as indicated by the RULA method (see Figure 1):

- Flexion-extension of spine, twisting and lateral bending;
- Flexion-extension of neck, twisting and lateral bending.

### 2.3. Ergonomic Risk Index Algorithm

**Figure 4:**
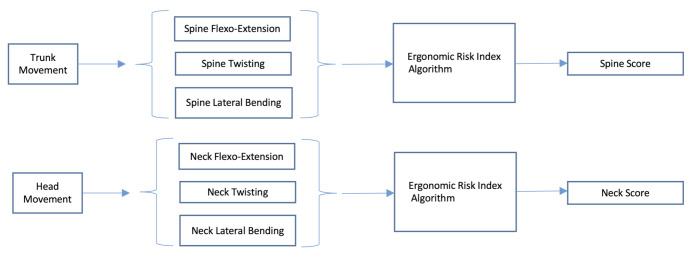
Spine and neck scores are obtained independently from the corresponding information. The same algorithm is applied for both segments. Both scores are entered in a look-up table to obtain an overall score for head and trunk as in the RULA approach.

The algorithm presented here processes the time course of neck and trunk segment angles to estimate a segment-specific score using a sliding time window approach. In a final stage, the scores are combined to obtain an overall time-varying score. The scoring procedure adopts a strategy mimicking the RULA approach. Specifically, angle values related to flexo-extension, twisting, and lateral bending of neck and trunk are quantized using segment-specific thresholds. A score ranging from 1 to 6, is obtained for each segment (see Figure 4). Both scores are then entered in the look-up table shown in Section 2.1 (see Figure 2) and merged with the information about legs and feet, to estimate an overall score for trunk and head. The information about legs and feet is set to 1 when legs and feet are in a supported and evenly balanced position, 2 otherwise. In this work, a constant value of 1 was set, taking into account the position of the surgeon during the whole procedure.

**Figure 5:**
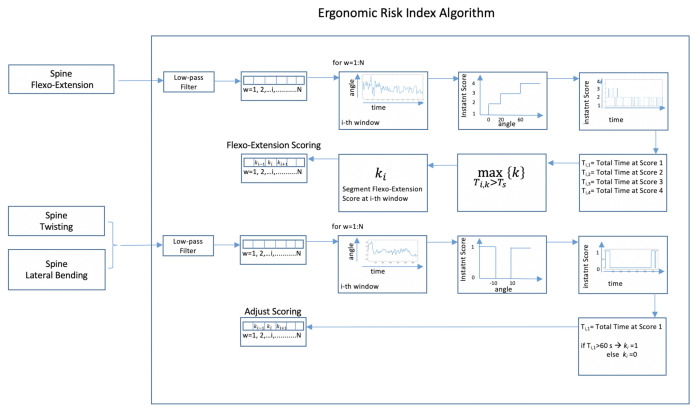
In this figure, the algorithm for estimating the scores related to each segment is shown. This algorithm is applied to neck and trunk: the thresholds applied to obtain the instant score, as shown, in the 4th block of each line, might vary for the two segments. In this case, the threshold values adopted for spine are shown. While the upper line is applied to flexo-extension, the lower line is applied for twisting and lateral bending, possibly resulting in an adjustment score ranging from 0 to +2.

In Figure 5, the algorithm used to estimate the scores of each section, i.e., head or trunk, is shown. The upper line of the scheme represents the scoring of the flexo-extension angle of the segment taken into account. A sliding time window approach is adopted, and this processing line ends with a score ranging from 1 to 4 for each of the N time windows. The processing steps represented by the lower line in Figure 5 are applied to detect any twisting or bending of the body segment considered and adjust the segment scoring related to flexo-extension. In fact, the RULA approach suggests that twisting and/or bending of trunk or neck should be taken into account, since they increase the risk level. For this category of movements, the maximum score to be added is equal to 1. The score of each segment can thus assume values between 1 and 6, considering the integration of the flexo-extension score (from 1 to 4) with the 2 extra points related to possible twisting or bending movements (see Figure 1). The scores obtained for the spine and neck are then entered in the look-up table (see Figure 2), along with the leg and feet supporting information, which in the considered scenario is a constant equal to 1, to get the overall score for head and trunk.

Now, the different steps that characterize the algorithm will be described. The first step pertains to the reduction of the effect of short time intervals characterized by abrupt angle changes. This is achieved by low-pass filtering the raw sensor signals, i.e., the raw angle time series, using a moving average filter with a time length of approximately 10 s. The low-pass filter cut-off frequency is thus equal to 0.038 Hz. The filtered signal is then divided into 5 min long time windows. The angle values related to each time step are then quantized by adopting different rules for the different segments and bending directions. This operation is shown by the fourth block present in both processing lines of Figure 5 that specifies the input-to-output mapping relationship used for the quantization operation. Regarding flexo-extension angles of both body segments, a staircase shape for the input-output relationship is chosen, as shown by the 4th block of the upper processing line in Figure 5. The threshold values are 0, 20, and 60 degrees for the spine and 0, 10, and 20 degrees for the neck. For both twisting and bending movements, the same approach is followed. Specifically, the processing of each movement’s information will result in a value equal to 1 when the corresponding angle is outside the interval [−10,+10] degrees. As a result of the above-described operations, the angles related to the three movements are mapped into a discrete and reduced set of values, which are the assigned scores at each time point. After the 4th block, specific strategies are proposed to provide a synthesis score for each 5 min long time window and each body segment, which will be the input of the look-up Table reported in Figure 2. For the flexo-extension case, the output score is the highest score that is kept for a total time longer than a given time threshold. The total time at each score is obtained by summing all the time intervals at that score. This strategy is depicted by the 5th block, which is used to estimate the total time spent for each score, and by the 6th block, which performs the score selection according to the above-described strategy. In this work, the time threshold was set equal to 1 min. As regards the twisting and bending cases, one was assigned when the corresponding angles were outside the chosen angle range, for a time longer than 1 min (see the 5th block in the lower processing line). The scores for spine and neck were estimated as the sum of flexo-extension scores and twisting and lateral bending adjustments and were then entered in the Table reported in Figure 2 to get the overall score for trunk and head.

### 2.4. Experimental Setup

**Figure 6:**
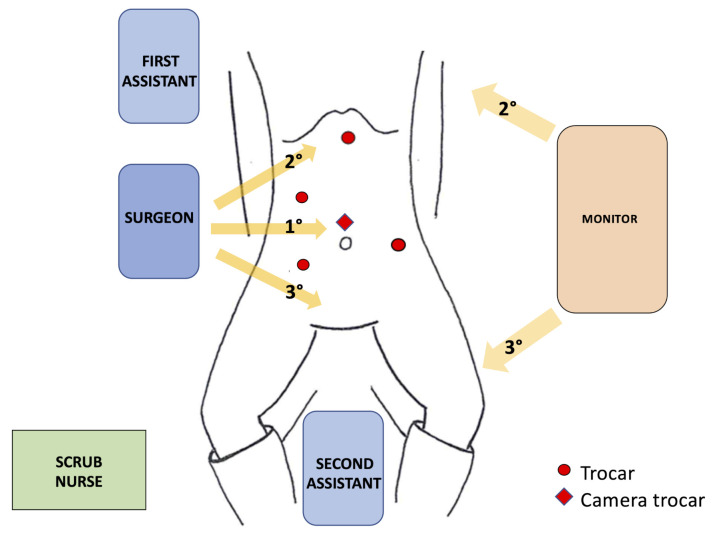
Operatory room setup. The patient is positioned on stirrups. Yellow arrows indicate the visual line of the surgeon during the three parts of the laparoscopic steps of the procedure with the consensual adjustment of the monitor.

To test the developed platform, we asked a surgeon to use the monitoring system during a real laparoscopic surgery operation. The surgeon is a 43 years old male who has been in the Unit of General and Emergency Surgery for 14 years. The operation chosen was the anterior resection of the rectum, which is performed frequently and is a well-standardized intervention lasting about 3 h, a duration compatible with the autonomy of the IMUs selected for this study. The Operatory Room (OR) setup is presented in Figure 6. The surgeons could adjust the monitor height and orientation, as well as the height, inclination, and lateral rotation of the patient bed. The surgical operation could be split into two main phases: the first laparoscopic procedure, which represents the longest portion of the intervention, and an open step (which is called the “mini-laparotomy procedure”) during which the surgeon performs a small incision on the supra-pubic area.

Moreover, during all the sessions, a camera was used to record the surgeon’s activity, in order to have a reference video on the variations in posture and on specific surgical steps. This solution allows identifying the possible interactions among the team components such as the surgeon’s request for new tools to the scrub nurse or the modification of the operating table position. All of these situations involve substantial changes to the surgeon’s posture and could be easily verified, labeled, and eventually excluded from the analysis. In this work, we decided to focus on time intervals when the surgeon was performing the laparoscopy or mini-laparotomy procedures. Moreover, the analysis was limited to the consecutive time intervals when the data flow from the IMUs was stable and without missing segments.

## 3. Results

**Figure 7:**
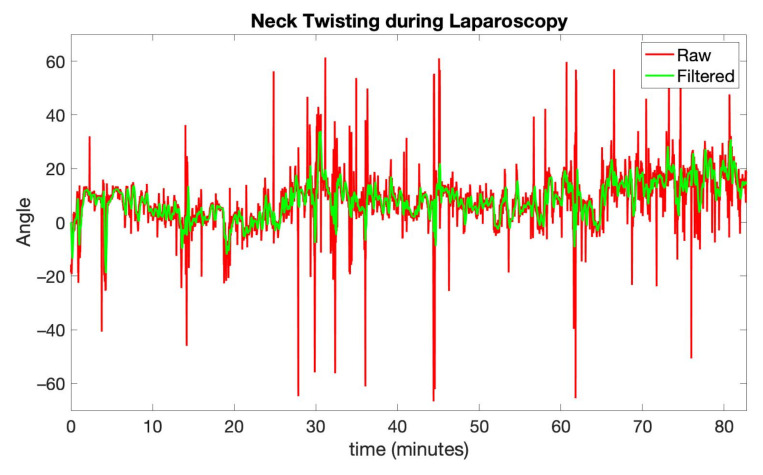
Raw and filtered signal related to neck twisting during laparoscopy.

The analysis was performed on two data blocks: the first one is related to the laparoscopy procedure, while the second one is related to the mini-laparotomy procedure. The time blocks do not contain the initial preparatory steps performed by the surgeon including the approach to the patient, the completion of the OR setup, and the positioning of the trocars. Moreover, the analysis of posture during the surgical procedure’s final steps, such as those related to the removal of the trocars, fascial, and skin closures, was not considered. The time duration was approximately 83 min for the laparoscopy part and 15 min for the mini-laparotomy section. In Figure 7, the raw signal related to neck twisting during laparoscopy is shown, along with the filtered signals. The results of the low-pass filtering operation, which was applied by using a moving average filter for the raw signals, are clearly visible. Specifically, the comparison between the filtered and raw signals in Figure 7 highlights how the positions that are held for a short time are smoothed so that their influence on the final score is reduced. In this recording, the raw signal presented abrupt angle changes, shorter than 3 s and larger than 30 degrees, which were completely removed by the low-pass filtering step.

**Figure 8:**
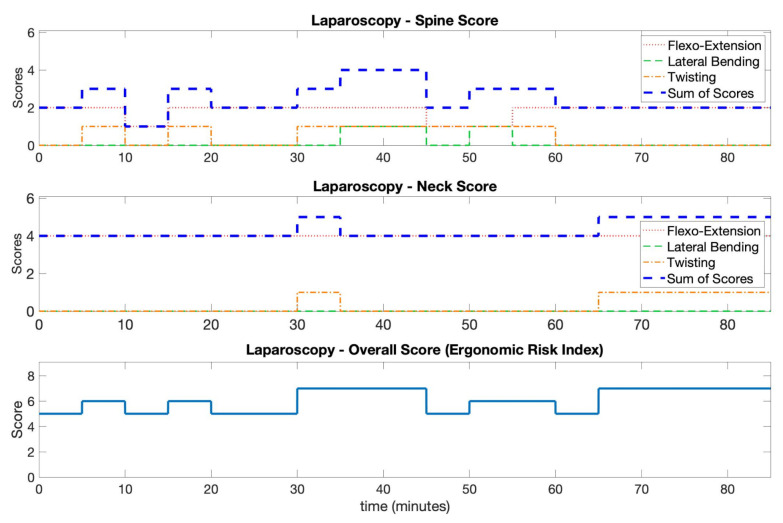
In this figure, the scoring results of the laparoscopy procedure are reported. The partial and total scores for the trunk and head, as well as the final score, obtained using the table in Figure 2, are shown.

**Figure 9:**
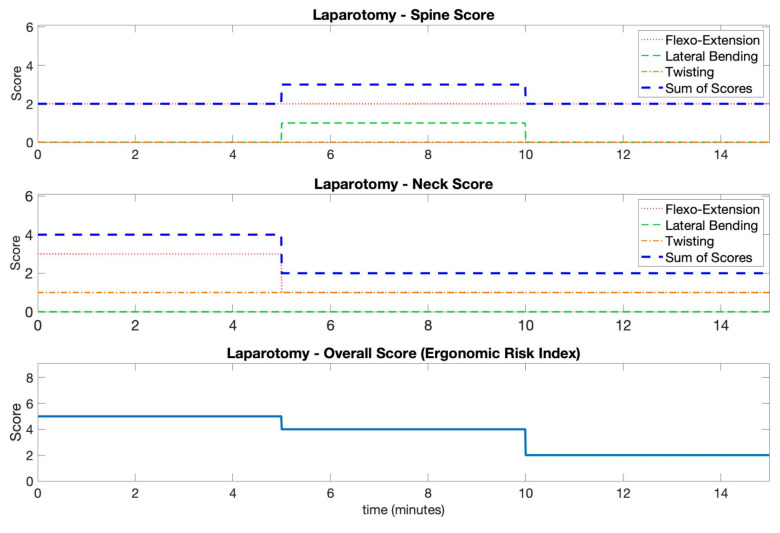
In this figure, the scoring results of the laparotomy procedure are reported. The partial and total scores for the trunk and head, as well as the final score, obtained using the table in Figure 2, are shown.

In the two upper axes of Figure 8, the partial and total scores for the neck and spine segments obtained during the laparoscopy procedure, i.e., the ergonomic risk indexes, are shown, while in Figure 9, the same scores are shown for the mini-laparotomy procedure.

The scores pertain to 5 min long windows and were obtained using the angle information at finer temporal scales. The partial scores describing flexo-extension, bending, and twisting for each segment are shown. These figures allow explaining how the partial and total scores were obtained, thus highlighting the possible concurrent contribution of the three angle components. For each procedure, the total scores of each segment were used to get an overall index by using the look-up table reported in Figure 2). The final results for the two procedures are shown in the bottom axes of Figure 8 and Figure 9.

**Figure 10:**
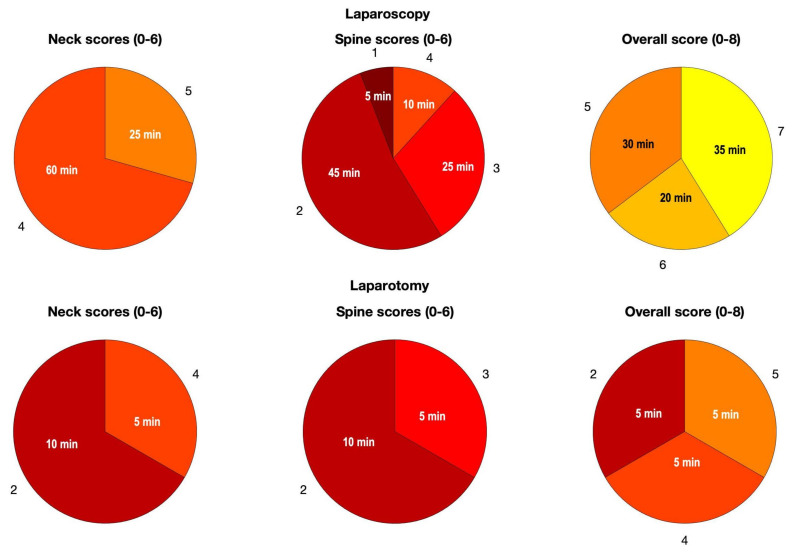
Pie charts describing the temporal percentages of the scores during the two procedures.

In Figure 10, the time percentages related to each score, for neck and spine segments, as well as for the ergonomic risk indexes are graphically depicted for the two procedures. The length, in minutes, of the overall time interval during which a score was obtained is shown as well.

## 4. Discussion

In this paper, we introduced and described the functionalities of a wearable sensor platform designed to monitor surgeon posture during surgery.The results from a surgical procedure were included in this paper as a case study to better introduce the platform. The proposed system could give relevant information within the more general assessment procedure performed with the RULA. In fact, an overall score for head and trunk equal to or greater than seven was observed for long intervals during the laparoscopic procedure. These scores are associated with a RULA total score equal to or greater than five (see Table C in [23]), thus indicating the need for a change in the operating conditions. The system highlighted that the surgeon maintained peculiar static positions of the body for an extended period of time. Noticeably, neck was kept in an extended position for the entire duration of the intervention. This can be seen by the flexo-extension component of the neck score equal to four in Figure 8. Neck extension is a typical problem related to screen positioning with respect to the surgeon’s head [36]. Since the surgeon has to look at the monitor, it is possible that a better setup of the operating room could have reduced this issue. As regards the spine position, a combination of flexion-lateral inclination and/or rotation was observed for more than 45 min. The data on the laparotomy procedure were related to a shorter observation. The estimated values of the ergonomic risk index were less critical. Most of the postural position could be attributed to prolonged flexion of both neck and spine, given that the surgeon has to look down. A twisted position of neck was observed during all the recording, and a concurrent lateral bending of spine was observed in a central period of the laparotomy procedure. The possibility of evaluating separately different body segments is very useful for the evaluation of the overall score and for the planning of behavioral interventions.

Our choice to use a scoring system derived from the RULA method to provide an ergonomic evaluation of the surgeon revealed some limitations present in this type of approach. In fact, one weakness found was the use of the same basic calculations applied to all the anatomical areas considered for the extraction of the risk index. As an example with systems such as the one we proposed for the evaluation of the lumbar area, since back pain is one of the most common MSDs in the world ([1,37]), it could be more efficient to provide more specific information on the movements performed by this body segment, such as the lateral speed of trunk and the times of permanence in maximum load. With these aspects in mind, our system can be easily modulated to optimize the score for specific purposes. In particular, various parameters (e.g., duration of the time window, threshold values) can be adjusted to give more weights to some positions or postures of the body segments.

Furthermore, another critical condition of the RULA method is represented by the fact that for the evaluation of the torsion or lateral flexion, angular thresholds must be set at the complete discretion of the developer. In our implementation, we set the following: when the body segment exceeded an twisting/bending angle greater than 10 degrees for a time interval greater than 60 s, a score of one was added to the overall risk index. Other angle thresholds could be chosen. In our case, the 10 degree threshold was three times the precision of the IMUs, thus allowing having a robust indication of bending or twisting postures.

Another characterizing point of the RULA tool is that the local and global scores are based on the analysis of the worker’s static postures, not taking into account the influence of the overall time spent in each evaluation interval. In fact, the ability of our instrument to record the temporal dynamics of the angles assumed by the different anatomical areas could allow a more effective evaluation of the ergonomic risk. In principle, IMU-based systems such as the one we presented could allow the development of new assessment tools taking into account the dynamics of score variation throughout the time span of the surgeon’s activity. In this pilot study, we decided to estimate the total time assigned to a given score in each time window. The final score was the highest among those awarded for more than 1 min. Obviously, other strategies are possible, either by taking into account more complex dynamics between the scores or by simply checking for homogeneous blocks of time.

A limitation of this study is that we could not estimate the overall RULA score, since some information was missing. Specifically, as regards the score we provided about trunk, head, and neck, the information related to muscle activity or the force/load score was not considered. According to the original RULA assessment, the former should be set to one when movements are repeated more than four times per minute, or when static postures are held for more than 1 min. The latter should be set to zero when intermittent forces or loads smaller than 2 kgfare present and take larger values, with a maximum equal to three, for larger forces or loads. More importantly, our system does not take into account the upper limb score.

One relevant step in the proposed approach is the design of the low-pass filter applied to the raw IMU outputs. Specifically, the low-pass filtering operation allows the reduction of the rapid movements that do not negatively affect the ergonomics of the surgical procedure. Moreover, it can be used to highlight the body positions that are kept for a longer time. In this study, we showed the effect of a low-pass filter with a large transition band, so that the changes at frequencies higher than 0.038 Hz were not abruptly suppressed. This allowed us to partially take into account the effect of big changes in body segment position. The cut-off frequency of the low-pass filter could be changed to differently weight the contribution of the time interval over which a position is held. This would allow highlighting specific dynamic features that are thought to be relevant for the worsening of musculoskeletal pathology.

The information describing legs and feet support was chosen to be a constant equal to one. This assumption can be modified, and the information about legs and feet can be estimated dynamically. In fact, during the surgical procedure, we noticed that the surgeon could change the distribution of her/his weight between the legs. In a future development, this information could be achieved by adding force sensors under feet or by estimating a model of leg balance using available sensors.

The information of bending, flexo-extension, and twisting of spine and neck was obtained using three IMU sensors, which guaranteed the advantages of the ability to detect movement directly at the site of interest, e.g., the operating room, and did not interfere with the worker’s activities. However, the use of these sensors could expose the evaluation to possible errors due to the positioning of the sensors themselves, to their movement due to both the sliding on skin, and to the sliding of skin over the skeleton. Future work could exploit the current system design, while improving the algorithm, to take into account a finer analysis of the IMUs’ output. Specifically, this could allow improving the actual scoring procedure by estimating the muscle activity index related to static postures or movement repeatability.

Another improvement could be related to the monitoring of upper limbs. This would necessitate a significant change of the system design with the use of further sensors, integrating current information with the electromyographic signal from trapezius, biceps, deltoid, and radial flexor carpal muscles [38]. Although this upgrade could be relevant for the chosen scenario, a careful analysis of the ergonomics of the system should be introduced, since it would involve a sensorization of upper arm and of wrist. This integrated EMG-IMU approach was not possible in this study to avoid the risk of overloading the surgeon with wearable tools during a real surgery. Having performed these assessments during a real surgery, rather than through simulations, represents a strength of the study: however, it was considered risky, as it could cause some discomfort to the surgeon during the surgery itself.

## 5. Conclusions

In this work, an innovative system for the assessment of surgeon posture was described and preliminary tested. The proposed system, used during a real surgical operation, was able to evaluate the ergonomic conditions of the surgeon by analyzing the signals of three IMU sensors attached to her/his body. To this end, an algorithm to estimate an ergonomic risk index was developed, borrowing relevant features of the well-established RULA approach. The proposed system automatically calculates a score related to the actual positions taken by the surgeon’s selected body segments. The main idea of this work was to develop a tool capable of indicating possible dangerous movements or postures assumed by surgeons to help them avoid the onset of musculoskeletal pathologies. The proposed approach could be adopted both during a training phase, to identify the subject-specific habits, as well as during the real surgical procedure to promptly alert the surgeon. Several parameters of the proposed scoring system can be modulated to highlight specific postures or body segment dynamics that are considered harmful. For this reason, the ability to evaluate the temporal dynamics of the movements assumed by the surgeons represents a further innovative element of the proposed system that could allow the development of a new ergonomic risk scoring system based not only on the static analysis of the operator’s posture.

## Data Availability

Data are available on request.
